# Assessment of the physical and chemical properties of vaginal discharge for the diagnosis of endometritis in dairy cattle

**DOI:** 10.14202/vetworld.2025.1322-1332

**Published:** 2025-05-25

**Authors:** Nest Dale Bartolome, Ruchikon Jongsuwanwattana, Sariya Asawakarn, Siriwat Suadsong, Kiattisak Sangpradit, Theerawat Swangchan-Uthai

**Affiliations:** 1 Department of Obstetrics, Gynecology and Reproduction, Faculty of Veterinary Science, Chulalongkorn University, Bangkok 10330, Thailand; 2 Center of Excellence in Animal Fertility Chulalongkorn University (CU-AF), Chulalongkorn University, Bangkok 10330, Thailand; 3 Department of Veterinary Physiology, Biochemistry Unit, Faculty of Veterinary Science, Chulalongkorn University, Bangkok, Thailand; 4 Department of Agricultural Engineering, Faculty of Engineering, Rajamangala University of Technology Thanyaburi, 39 Moo 1, Rangsit-Nakhonnayok Road, Thanyaburi 12110, Thailand

**Keywords:** bovine endometritis, cow-side diagnosis, leukocyte esterase strip, pH meter, postpartum reproductive health, vaginal discharge analysis

## Abstract

**Background and Aim:**

Postpartum endometritis is a major cause of infertility in dairy cattle, impacting herd productivity and economic sustainability. Although traditional diagnostic methods are available, there remains a need for simple, rapid, and accurate cow-side diagnostic tools to facilitate early detection and management of clinical endometritis (CE) and subclinical endometritis (SCE). This study aimed to (i) evaluate the diagnostic performance of vaginal discharge parameters - pH, total dissolved solids (TDS), salinity, and electrical conductivity (EC) - using a portable flat-surface electrode probe, and pH and leukocyte esterase (LE) activity using reagent strips and (ii) establish optimal cut-off values for these parameters to support practical on-farm screening of endometritis.

**Materials and Methods:**

Fifty-eight postpartum Holstein Friesian cows were enrolled. Vaginal discharge was collected using a Metricheck device and analyzed for pH, TDS, salinity, and EC. Concurrently, pH and LE activity were assessed using reagent strips. Endometrial cytology was performed to diagnose CE and SCE. Diagnostic performance was evaluated using receiver operating characteristic (ROC) curve analysis.

**Results:**

pH values measured by both the meter and strip were significantly higher in cows with normal uterine health compared to those with CE (p = 0.010 and p = 0.008, respectively). LE activity was significantly elevated in cows with CE (p = 0.001). ROC analysis identified optimal cut-off values: pH meter ≤8.35 (area under the curve [AUC] = 0.768) and LE strip ≥2 (AUC = 0.835) for diagnosing CE. Combining pH and LE strip results improved diagnostic performance (AUC = 0.801), achieving 65.22% sensitivity, 87.5% specificity, and 76.6% accuracy. TDS, salinity, and EC were not significantly associated with uterine health status (p > 0.05).

**Conclusion:**

The combined evaluation of vaginal discharge pH and LE activity offers a practical, cost-effective cow-side screening method for diagnosing endometritis in dairy cattle. In contrast, TDS, salinity, and EC measurements were not diagnostically informative. The proposed approach may enhance herd health management by enabling timely identification and treatment of endometritis.

## INTRODUCTION

Postpartum reproductive diseases, particularly endometritis, which cause infertility or subfertility, pose a significant economic threat to livestock production by reducing animal productivity and performance. Early detection of these disorders is essential for preserving the profitability of the livestock industry and safeguarding food security [1, 2]. Bovine endometritis is generally classified into clinical endometritis (CE) and subclinical endometritis (SCE), with each presenting distinct diagnostic challenges [3, 4]. Both CE and SCE prolong the interval from calving to first insemination, increase the number of services per conception, and negatively impact milk yield and culling rates [5, 6]. Selecting appropriate diagnostic tools for uterine diseases is critical for timely therapeutic decision-making. Early screening enables the identification of at-risk cows, facilitating timely intervention and mitigating the severity of symptoms [7]. Field diagnosis of endometritis remains challenging due to the lack of simple, rapid, and reliable techniques [8]. Traditional diagnostic methods for CE, such as clinical evaluation, rectal palpation, ultrasonography, and vaginal discharge analysis, rely heavily on the detection of purulent vaginal discharge (PVD) [9]. However, reliance on PVD alone can result in diagnostic inaccuracies, as similar discharges may arise from vaginitis or cervicitis [10]. Tools such as the Metricheck device assist in the collection of mucus for visual assessment; however, additional tests, including endometrial cytology, are often required for definitive diagnosis [11, 12]. The diagnosis of SCE is even more challenging, as it is largely asymptomatic and often remains undetected in commercial herds due to the absence of reliable on-farm tests [13]. More sophisticated diagnostic techniques, including endometrial cytology and histological examination by biopsy, are necessary to confirm SCE [14]. Endometrial cytology remains the gold standard for diagnosing both CE and SCE, with elevated polymorphonuclear neutrophil (PMN) counts serving as a principal indicator of endometrial inflammation [10]. CE is characterized by the presence of PVD, which can be graded based on the characteristics of the vaginal mucus, whereas endometrial inflammation is diagnosed through cytology (>5% PMN) [15, 16]. In contrast, SCE does not present visible PVD and is diagnosed predominantly through endometrial cytology (>5% PMN) [17, 18]. Furthermore, Madoz *et al*. [19] proposed PMN cut-off thresholds adjusted according to days in milk (DIM) to enhance SCE diagnostic precision: 8% for 21–33 DIM, 6% for 34–47 DIM, 4% for 48–62 DIM, and an overall threshold of 5% for 21–62 DIM. Despite the diagnostic accuracy of these methods, their practical application for routine on-farm use remains limited [20].

The implementation of rapid screening tests for endometritis is crucial not only for selecting appropriate therapeutic strategies but also for ensuring timely management decisions regarding overall herd health. Recent research has explored alternative diagnostic approaches focused on the physicochemical properties of vaginal discharge, which remain underutilized for detecting both CE and SCE. Vaginal discharge may reflect the reproductive health and fertility status of the animal [21], serving as a valuable diagnostic and prognostic marker throughout the bovine reproductive cycle [22]. Previous studies by Srinivasan *et al*. [23] and Li *et al*. [24] have indicated that certain components of vaginal discharge, such as cytokines and chemokines, could act as early markers for uterine disorders shortly after calving or even before clinical signs become apparent. This strategy offers the potential for the development of non-invasive, rapid diagnostic tools based on vaginal discharge analysis. In this context, we hypothesized that the characteristics and properties of cows’ vaginal discharge - specifically, pH, leukocyte esterase (LE) activity, salinity, total dissolved solids (TDS), and electrical conductivity (EC) - could offer critical insights into the uterine health of postpartum dairy cows. Vaginal discharge pH has been extensively studied, particularly in relation to fertility status [25]. Other physicochemical parameters, including TDS, salinity, and EC, typically evaluated in water quality assessments, could also be applied to vaginal discharge analysis, given that it comprises 92%–95% water and ions and 5%–8% dissolved solids [26]. In addition, vaginal discharge contains mucin, glycoproteins, lipids, and salts such as NaCl, KCl, and CaCl^2^ [27]. Sodium chloride (NaCl), the predominant salt in vaginal discharge, contributes to its ionic strength and may serve as a potential marker for the early detection of endometritis [25].

Despite significant advances in the diagnosis of postpartum endometritis in dairy cattle, the present clinical practices remain hindered by the absence of simple, rapid, and reliable cow-side diagnostic tools suitable for both CE and SCE. Traditional diagnostic approaches, such as endometrial cytology and histopathology, although highly accurate, are invasive, labor-intensive, and impractical for routine on-farm application. While the detection of PVD through visual inspection is widely used, its specificity is limited, and it fails to reliably detect subclinical cases. Emerging evidence has suggested that certain physicochemical properties of vaginal discharge, such as pH, TDS, salinity, EC, and LE activity, may serve as potential surrogate markers for uterine inflammation. However, previous studies have predominantly focused on uterine or cervical samples, with limited validation of vaginal discharge properties as practical diagnostic indicators. Furthermore, the diagnostic performance of combining multiple physicochemical markers has not been systematically evaluated, leaving a critical gap in the development of rapid, non-invasive, and field-applicable diagnostic strategies for endometritis.

Therefore, this study aimed to evaluate the diagnostic utility of vaginal discharge parameters - specifically pH, TDS, salinity, and EC measured by a portable flat-surface electrode probe, as well as pH and LE activity assessed by reagent strips – in relation to CE and SCE in dairy cattle. In addition, the study sought to determine optimal cutoff values for these parameters, individually and in combination, to establish a practical, cow-side screening approach for the early and accurate detection of endometritis under field conditions.

## MATERIALS AND METHODS

### Ethical approval

All procedures involving animals in the experiment were conducted in accordance with the guidelines and regulations set by Institute for Animal Care and Use Committee of Faculty of Veterinary Science, Chulalongkorn University (protocol No. 2231045, dated November 6, 2022).

### Study period and location

The study was conducted from June 2023 to March 2024 at the Chulalongkorn University Dairy Research Farm (Saraburi) and the Animal Hospital and Student Training Center, Faculty of Veterinary Science, Chulalongkorn University (Nakorn Pathom, Thailand).

### Animals

Fifty-eight Holstein Friesian cows (mean age: 38.12 ± 14.48 months; parity: 1^st^–3^rd^ lactation; DIM: 37 ± 14.30) were enrolled from the Chulalongkorn University Dairy Research Farm (Saraburi) and the Animal Hospital and Student Training Center, Faculty of Veterinary Science, Chulalongkorn University (Nakorn Pathom, Thailand). All cows were clinically healthy, exhibiting no signs of systemic illnesses such as mastitis or ketosis, and had not received antibiotic treatment within 30 days before enrolment. Cows with a history of chronic reproductive disorders, including uterine prolapse or retained placenta, were excluded. Body condition scores ranged from 2.25 to 3.75. Animals that experienced dystocia or required assisted calving were also excluded. Reproductive examinations, including inspection for PVD, rectal palpation, and ultrasonography of the ovaries and uterus, were performed during routine monthly veterinary visits starting at 15 DIM.

To account for possible variations related to reproductive cycle stages, cows were categorized according to ovarian activity at the time of sampling. Cows presenting a corpus luteum (CL) on transrectal ultrasonography were classified as being in the luteal phase, while cows lacking a CL were classified as being in the follicular phase. Subsequently, the physicochemical parameters (pH, TDS, salinity, and EC) and reagent strip results (pH and LE activity) were compared between the two groups.

### Group allocation

Cows were allocated into three groups based on a combination of visual vaginal discharge scoring (VDS) and endometrial cytology results: Normal uterine health, CE, and SCE. The group allocation followed a non-randomized, observational design. A single trained observer performed all VDS assessments and endometrial cytology analyses to ensure consistency. VDS evaluation and endometrial cytology were conducted independently under blinded conditions. Vaginal discharge samples were collected using a Metricheck™ device (Simcro Limited, Hamilton, New Zealand) and transferred into sterile containers. Vaginal discharge characteristics were analyzed using two diagnostic tools:

1. An Extech EC500 ExStik® II probe (Ponpe Instruments, Thailand) to measure pH, TDS, EC, and salinity
2. Combur10 Test® reagent strips (Roche Diagnostics, Mannheim, Germany) to assess pH and LE activity.

Receiver operating characteristic (ROC) curve analyses were conducted to determine the optimal diagnostic cut-off values.

### Vaginal discharge collection and visual scoring

Vaginal discharge samples were collected in the morning after milking to standardize collection timing across animals. Before sampling, the perineum and external genitalia were washed with water and wiped dry with a clean towel to minimize contamination. The Metricheck device was inserted into the vulva and advanced toward the cranial vagina. Following gentle elevation and caudal retraction of the device, the collected discharge was classified based on a four-point VDS scale [28]:

- Score 0: Clear discharge
- Score 1: Discharge with flecks of white to off-white pus
- Score 2: Mucopurulent discharge containing <50% white to off-white pus
- Score 3: Purulent discharge containing >50% white to yellowish pus.

Cows with a VDS ≥2 were considered positive for PVD. A minimum of approximately 1 mL of vaginal discharge was collected per cow to ensure sufficient volume for analysis. All samples were analyzed within 5-min post-collection.

### Measurement of vaginal discharge parameters

### Reagent strip analysis

Collected vaginal discharge samples were applied directly onto Combur10 Test® reagent strips (Roche Diagnostics GmbH). The pH was recorded based on a colorimetric scale ranging from 6 to 9, and LE activity was scored as follows [29]:

- 0: Negative
- 1: + (approximately 10–25 leukocytes/μL)
- 2: ++ (approximately 75 leukocytes/μL)
- 3: +++ (approximately 500 leukocytes/μL).

The strips were evaluated by a trained observer using the manufacturer’s standardized color chart.

### Extech EC500 ExStik® II probe analysis

The Extech EC500 ExStik® II meter (EC500, Extech Instruments, Nashua, NH, USA) was used to measure pH, EC, TDS, and salinity according to the manufacturer’s instructions. After activating the device, the appropriate measurement mode was selected. The electrode was fully submerged in the vaginal discharge sample, ensuring the elimination of air bubbles by gently stirring the sample. The device automatically displayed readings for each parameter. The mode/hold button was pressed and held for 2 s to toggle between measurement units.

#### Endometrial cytobrush cytology

Endometrial cytology was performed using a cytobrush technique adapted from a previous study by Swangchan-Uthai *et al*. [30]. Two experienced veterinarians carried out the sampling. A modified guard swab uterine sampling tube – a long, hollow stainless-steel tube with a rounded end – was used to introduce a Cytobrush Plus® GT device (Medscan Medical AB, Kista, Sweden). After rectal evacuation and aseptic preparation of the perineal area, the sampling tube was inserted transrectally through the cervix into the uterine body. A sanitary sheath was utilized to prevent contamination during passage.

Upon reaching the uterine body, the cytobrush was deployed and rotated clockwise to collect endometrial cells. The cytobrush was then retracted and removed. Smears were prepared by rolling the brush onto clean glass slides (Sailbrand™ Sailbrand Ltd., South Yorkshire, UK), fixing them in 70% methanol, and staining with Diff-Quik® (Siemens Healthcare Diagnostics Inc., Tarrytown, NY, USA). Cytological evaluation was performed under light microscopy at 1,000× magnification, counting 200 cells per slide to determine the proportion of PMNs.

### Determination of uterine health status

The uterine health status of each cow was determined by combining VDS and endometrial cytology findings, following established criteria [17, 19, 31]:

- CE: Presence of PVD (VDS ≥2) and ≥5% PMN on cytology
- SCE: Absence of PVD but ≥5% PMN on cytology
- Normal uterine health: Neither PVD nor elevated PMN (>5%) present.

The ≥5% PMN threshold has been reported to provide high interobserver reproducibility [32, 33].

### Statistical analysis

All statistical analyses were conducted using SAS software version 9.4 (SAS Institute Inc., Cary, NC, USA). The Shapiro-Wilk W-test and Qualitative-Quantitative plots were employed to assess the normality of continuous variables. The cow was considered the experimental unit. Descriptive statistics were reported as mean ± standard deviation for animal-related parameters and mean ± standard error of the mean for vaginal discharge parameters.

General linear models (PROC GLM) were used to compare the pH, TDS, salinity, and EC values among the three groups, with least-square means compared using the least significant difference test. The Kruskal-Wallis test, followed by pairwise multiple comparisons, was used to analyze differences in pH strip readings and LE activity among groups.

Logistic regression analysis was performed by dichotomizing PVD, pH meter, pH strip, and LE strip results at various cut-off levels to classify cows as affected (CE or SCE) or healthy. Multiple parameter combinations (e.g., PVD/pH/LE) were also evaluated to determine whether they improved diagnostic performance. ROC curve analysis was applied to assess the diagnostic performance of individual and combined parameters against the gold standard (endometrial cytology). The area under the ROC curve (area under the curve [AUC]) quantified diagnostic accuracy, with values ranging from 0.5 (no discriminative ability) to 1.0 (perfect discrimination). A p < 0.05 was considered statistically significant.

Sample size calculation was conducted using G*Power software version 3.1 (Heinrich-Heine-Universität, Düsseldorf, Germany), applying a one-way analysis of variance with fixed effects. Assuming an effect size of 0.45, the study achieved an estimated power of 80%. For ROC analysis, sample sizes (e.g., CE, n = 13; normal uterine health, n = 27) provided approximately 85% power to detect a significant AUC of at least 0.80, confirming sufficient statistical robustness.

## RESULTS

### Effect of estrous cycle stage on vaginal discharge parameters

To assess whether the reproductive cycle stage influenced the measured vaginal discharge parameters, cows were categorized into two groups based on transrectal ultrasonography findings at the time of sampling: follicular phase (no CL; n = 21) and luteal phase (CL present; n = 16). Comparative analyses of pH (meter and strip), TDS, salinity, EC, and LE activity revealed no statistically significant differences between the two reproductive phases (p > 0.05). Thus, under the conditions of this study, the estrous cycle stage did not affect the physicochemical properties or LE activity of vaginal discharge.

### Uterine health status of the study population

Integration of VDS scoring and endometrial cytology identified 13 cows diagnosed with CE, 11 cows with SCE, and 27 cows with normal uterine health. Seven cows were excluded due to the inability to obtain cytological samples. Among the evaluated animals, 54% exhibited a VDS score of 0, 24% had a VDS of 1, 10% had a VDS of 2, and 12% had a VDS of 3. Regarding pH strip results, 2% of cows exhibited a pH of 6, 9% a pH of 7, 54% a pH of 8, and 31% a pH of 9. LE strip scoring showed that 12% of cows had a score of 0, 31% a score of 1, 40% a score of 2, and 9% a score of 3. Due to insufficient sample volume, some measurements could not be obtained from all cows. A summary of the collected observations is presented in Table 1.

**Table 1:** Summary of the data collected from the cows included in the study.

Parameter	Observations		

	Normal uterine health	SCE	CE
pH meter	25	9	10
TDS	14	7	9
Salinity	14	7	9
EC	13	7	9
pH Strip	26	10	13
LE	24	10	13

SCE=Subclinical endometritis, CE=Clinical endometritis, TDS=Total dissolved solids, EC=Electrical conductivity, LE=Leukocyte esterase

### Differences among uterine health groups based on single diagnostic parameters

The mean values of pH (meter and strip), TDS, salinity, EC, and LE activity for the three uterine health groups are summarized in Table 2. A significant difference in pH meter readings was observed across groups (p = 0.017). *Post hoc* multiple comparisons revealed that cows with normal uterine health exhibited significantly higher pH meter values compared to cows with CE (p = 0.010). In addition, cows with SCE had significantly higher pH meter values compared to those with CE (p = 0.012). However, no significant difference was observed between cows with normal uterine health and SCE.

**Table 2:** The parameters measured using the Extech EC500 ExStik® II probe from three different health groups are reported as least square mean ± SEM: pH meter, TDS, salinity, and EC. The pH strip and LE are reported as mean ± SD.

Instrument	Parameter	Normal uterine health	SCE	CE	p-value
Extech EC500 ExStik® II	pH meter	8.54 ± 0.62^a^	8.67 ± 0.30^a^	7.90 ± 0.84^b^	0.017
	TDS (ppt)	3.75 ± 0.47^a^	3.94 ± 0.66^a^	4.73 ± 0.59^a^	0.422
	Salinity (ppt)	3.58 ± 0.43^a^	3.12 ± 0.61^a^	3.90 ± 0.54^a^	0.636
	EC (mS)	5.27 ± 0.63^a^	5.72 ± 0.86^a^	6.69 ± 0.75^a^	0.365
Combur10 Test®	pH strip	8.38 ± 0.70^a^	8.40 ± 0.52^a^	7.61 ± 0.65^b^	0.005
	LE	1.25 ± 0.85^a^	1.50 ± 0.71 ^a^	2.31 ± 0.48^b^	0.0008

The different superscript letters in a row represent a significant difference. SEM=Scanning electron microscope, TDS=Total dissolved solids, EC=Electrical conductivity, CE=Clinical endometritis, SCE=Subclinical endometritis, SD=Standard deviation, LE=Leukocyte esterase

Similarly, analysis of pH strip readings demonstrated significant differences between groups (p = 0.005). Pairwise comparisons showed that cows with normal uterine health had significantly higher pH strip scores than those with CE (p = 0.008).

For LE strip readings, significant differences were also observed among the three groups (p = 0.0008). Cows with CE had significantly higher LE scores compared to cows with normal uterine health (p = 0.001) and SCE (p = 0.015). No significant difference in LE scores was found between cows with normal uterine health and those with SCE.

### Diagnostic performance based on ROC analysis

#### Single-parameter ROC analysis for CE detection

ROC analysis was performed to evaluate the discriminative ability of the PVD, pH meter, pH strip, and LE strip individually for the diagnosis of CE.

**Figure 1:**
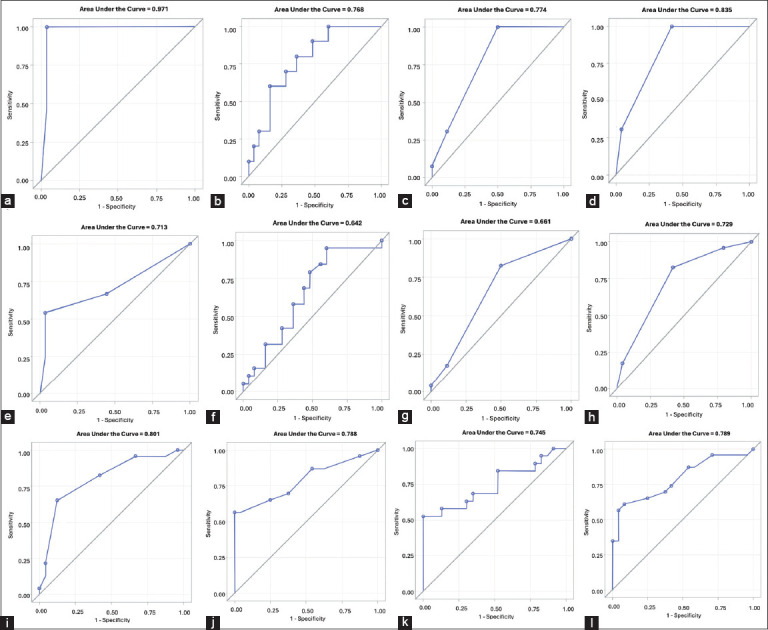
ROC curve of (a) PVD for CE, (b) PVD for combined CE and SCE pH meter for CE, (c) pH strip + LE strip for combined CE and SCE, (d) pH meter for CE, (e) pH meter for combined CE and SCE, (f) PVD + LE strip for combined CE and SCE, (g) pH strip for CE, (h) pH strip for CE, (i) PVD + pH meter + LE strip for combined CE and SCE, (j) LE strip for CE, (k) LE strip for CE, (l) PVD + pH strip + LE strip for combined CE and SCE against endometrial cytology as a diagnostic test for endometritis. ROC=Receiver operating characteristic, VDS=Vaginal discharge scoring, CE=Clinical endometritis, SCE=Subclinical endometritis, PVD=Purulent vaginal discharge, LE=Leukocyte esterase.

- The PVD with cut-off of ≥2 attained an AUC of 0.972 (95% CI: 0.91-1.00; p < 0.0001) 100% sensitivity, 96.30% specificity and 97.50% accu- racy (Table 3, Figure 1a).
- The pH meter yielded an optimal cut-off value of ≤8.35, achieving an AUC of 0.768 (95% confidence interval [CI]: 0.60–0.93; p = 0.001), with 70.0% sensitivity, 72.0% specificity, and 71.42% overall accuracy (Table 3, Figure 1c).
- The pH strip, with a cut-off of ≤8, demonstrated an AUC of 0.77 (95% CI: 0.60–0.90; p < 0.0001), sensitivity of 100%, specificity of 50%, and accuracy of 66.67% (Table 3, Figure 1d).
- The LE strip, with a cut-off ≥2, showed the highest single-parameter performance, with an AUC of 0.835 (95% CI: 0.73–0.94; p < 0.0001), sensitivity of 100%, specificity of 58.33%, and accuracy of 73.97% (Table 3, Figure 1e).

**Table 3:** Performance of vaginal discharge using single parameter PVD, pH, and LE strip for detecting CE in endometrial cytology.

Parameter	Disorders	AUC	Cut-off	TP	TN	FP	FN	ACC	Se	Sp	PPV	NPV
PVD	CE	0.972	≥2	13	26	1	0	97.5	100	96.30	92.86	100
pH meter	CE	0.768	≤8.35	7	18	7	3	71.42	70.00	72.00	50.00	85.71
pH strip	CE	0.77	≤8.0	13	13	13	0	66.67	100	50.00	50.00	100
LE strip	CE	0.835	≥2.0	13	14	10	0	73.97	100	58.33	56.50	100

The pH meter results were recorded using the Extech meter; the pH strip was recorded in four categories, 6–9, based on changes in color; the LE results were recorded in four categories: Negative was recorded as 0, + (∼10–25 Leu/μL) was recorded as 1, + + (∼75 Leu/μL) was recorded as 2, and + + + (∼500 Leu/μL) was recorded as 3. PVD=Purulent vaginal discharge, LE=Leukocyte esterase, CE=Clinical endometritis, AUC=Area under the curve, TP=True positive, TN=True negative, FP=False positive, FN=False negative, ACC=Accuracy, Se=Sensitivity, Sp=Specificity, PPV=Positive predictive value, NPV=Negative predictive value

#### Combined-parameter ROC analysis for CE and SCE detection

When CE and SCE groups were combined, the ROC analysis indicated:

- The PVD with cut off of ≥2 attained an AUC of 0.713 (95% CI:0.57-0.86; p = 0.0018 ), 54.17% sensitivity, 96.30 specificity and 76.47 accuracy (Table 4, Figure 1b).
- The pH meter cut-off of ≤8.69 achieved an AUC of 0.642 (95% CI: 0.47–0.81; p = 0.096), with 78.95% sensitivity, 52.0% specificity, and 65.31% accuracy (Table 4, Figure 1f).
- The pH strip at cut-off ≤8 showed an AUC of 0.66 (95% CI: 0.52–0.80; p = 0.024), sensitivity of 82.61%, specificity of 50%, and accuracy of 65.31% (Table 4, Figure 1g)
- The LE strip at cut-off ≥2 resulted in an AUC of 0.729 (95% CI: 0.596–0.86; p = 0.0007), with 82.61% sensitivity, 58.61% specificity, and 70.21% accuracy (Table 4, Figure 1h).

**Table 4:** Performance of vaginal discharge using combinations of parameter PVD, pH, and the LE strip for detecting combined CE and SCE groups in endometrial cytology.

Parameter	Disorders	AUC	Cut-off	TP	TN	FP	FN	ACC	Se	Sp	PPV	NPV
PVD	CE + SCE	0.713	≥2.0	13	26	1	11	76.47	54.17	96.3	92.86	70.27
pH meter	CE + SCE	0.64	≤8.69	15	13	12	4	63.63	78.95	52.0	55.56	76.47
pH strip	CE + SCE	0.66	≤8.0	19	13	13	4	65.31	82.61	50.0	59.38	76.47
LE strip	CE + SCE	0.729	≥2.0	19	14	10	4	70.21	82.61	58.33	65.52	77.78
pH meter + LE strip	CE + SCE	0.749	≤8.69 and ≥2.0	14	16	7	5	71.43	73.68	69.57	66.67	76.19
pH strip + LE strip	CE + SCE	0.801	≤8.0 and ≥2.0	15	21	3	8	76.6	65.22	87.5	83.33	72.41
PVD + pH meter	CE + SCE	0.705	≥2.0 and ≤8.69	10	24	1	9	77.27	52.63	96	90.91	72.73
PVD + pH strip	CE + SCE	0.732	≥2.0 and ≤8.0	13	25	1	10	77.55	56.52	96.15	92.86	71.43
PVD + LE	CE + SCE	0.788	≥2.0 and ≥2.0	13	24	0	10	78.72	56.52	100	100	70.59
PVD + pH meter + LE	CE + SCE	0.745	≥2.0, ≤8.69 and ≥2.0	10	23	0	9	78.57	52.63	100	100	71.88
PVD + pH strip + LE	CE + SCE	0.789	≥2.0, ≤8.0 and ≥2.0	14	22	2	9	76.6	60.87	91.67	87.5	70.97

PVD=Purulent vaginal discharge, LE=Leukocyte esterase, CE=Clinical endometritis, SCE=Subclinical endometritis, AUC=Area under the curve, TP=True positive, TN=True negative, FP=False positive, FN=False negative, ACC=Accuracy, Se=Sensitivity, Sp=Specificity, PPV=Positive predictive value, NPV=Negative predictive value

Among the combined-parameter models:

- The combination of pH strip and LE strip yielded the highest diagnostic performance, with an AUC of 0.801 (95% CI: 0.673–0.929; p < 0.0001), sensitivity of 65.22%, specificity of 87.5%, and overall accuracy of 76.6% (Table 4, Figure 1i).
- The PVD and LE strip combination demonstrated an AUC of 0.788 (95% CI: 0.654–0.922; p < 0.0001), with a sensitivity of 56.53%, specificity of 100%, and accuracy of 78.72% (Table 4, Figure 1j).
- Combining PVD, pH meter, and LE strip resulted in an AUC of 0.745 (95% CI: 0.584–0.905; p = 0.0028), with a sensitivity of 56.63%, specificity of 100%, and accuracy of 78.57% (Table 4, Figure 1k).
- The combination of PVD, pH strip, and LE strip achieved an AUC of 0.789 (95% CI: 0.656–0.922; p < 0.0001), sensitivity of 60.87%, specificity of 91.67%, and accuracy of 76.6% (Table 4, Figure 1l).

### Notes on measurement categorization

- pH meter readings were obtained using the Extech EC500 m
- pH strip readings were categorized based on color changes from 6 to 9
- LE strip results were categorized as 0 (negative), 1 (+, approximately 10–25 leukocytes/μL), 2 (++ ~75 leukocytes/μL), and 3 (+++ ~500 leukocytes/μL).

## DISCUSSION

### Utility of vaginal discharge analysis for diagnosing endometritis

The findings of this study highlight the potential utility of vaginal discharge analysis, particularly through pH measurement using a flat-surface electrode probe and the assessment of pH and LE activity through Combur10 Test® reagent strips (Roche Diagnostics GmbH), for the diagnosis of endometritis. In contrast, TDS, salinity, and EC demonstrated insufficient diagnostic performance. To the authors’ knowledge, this is the first study employing a flat-surface electrode probe to differentiate cows with normal uterine health, SCE, and CE based on vaginal discharge samples.

### Vaginal pH assessment and its diagnostic value

In this study, vaginal pH was assessed using two distinct devices: A pH meter and a pH strip. Significant differences were observed in pH measurements between cows with normal uterine health and those with CE, as well as between SCE and CE groups. Optimal cut-off values for distinguishing normal uterine health from CE were identified as ≤8.35 for the pH meter and ≤8.0 for the pH strip. Similarly, for combined CE and SCE, the cut-offs were ≤8.69 (m) and ≤8.0 (strip).

A general trend toward alkaline pH was observed across all groups, with cows exhibiting normal uterine health having higher mean pH values than cows with CE. These results align with those of Modi *et al*. [34], who reported a mean pH of 8.39 ± 0.17 for cows with normal uterine health, compared to 6.19 ± 0.18 in cows with endometritis. This trend suggests that a more alkaline vaginal environment is associated with improved reproductive health. Supporting this, prior studies noted that a vaginal pH >8 during estrus is associated with higher conception rates [21], and a cervicovaginal mucus pH ≥8.30 correlated with higher pregnancy rates compared to pH ≤7.76 [35].

However, contrasting findings exist. Previous studies by Cheong *et al*. [36], Bedewy and Rahaway [37], Raval *et al*. [38], and Parikh *et al*. [39] reported higher pH values in cows with endometritis based on uterine lavage and cervical discharge samples, with proposed cut-offs ≥7.0 for diagnosis. Such discrepancies may be attributed to differences in sample type and sampling methods. Unlike these studies, which evaluated cervical or uterine samples, the present study directly assessed vaginal discharge, possibly reflecting a localized inflammatory response more accurately. Furthermore, bacterial metabolites and inflammatory exudates in cows with CE may alter vaginal discharge pH, contributing to the alkaline shift observed [28, 40].

Other factors, including the measurement method, estrogen levels, and estrous phase, could also influence vaginal pH [41, 42]. Although not directly evaluated, the presence of a CL in most cows could have contributed to the higher alkaline pH observed. Tsiligianni *et al*. [43] previously demonstrated dynamic pH fluctuations across the estrous cycle, with pH reaching its lowest point at the end of estrus and rising post-ovulation.

### Diagnostic performance of TDS, salinity, and EC

Measurements of TDS, salinity, and EC across uterine health groups did not differ significantly. However, trends were observed, with cows with CE displaying the highest TDS, salinity, and EC levels. These findings are consistent with a previous study by Van Schyndel *et al*. [44] that reported limited diagnostic accuracy of TDS measurement using low-volume uterine lavage samples.

Variations in salinity and EC may be explained by mucus volume fluctuations and the presence of purulent material, both of which are influenced by hormonal and health status changes [45, 46]. NaCl, the predominant salt in cervical mucus, contributes to its ionic strength and has been linked to the fern pattern phenomenon during crystallization [47–49]. Given that vaginal discharge is a hydrogel primarily composed of water, salts, mucin-type glycoproteins, proteins, amino acids, and lipids [50–52], these compositional factors may account for the observed trends without achieving statistical significance.

### Diagnostic potential of LE activity

LE strip testing emerged as a promising cow-side diagnostic tool for endometritis. Significantly elevated LE activity was observed in cows with CE compared to those with SCE or normal uterine health. The optimal LE cut-off value (≥2) provided effective discrimination in both CE alone and combined CE/SCE groups.

These findings are consistent with previous studies by Hajıbemanı *et al*. [29], Cheong *et al*. [36], Van Schyndel *et al*. [44], Santos *et al*. [53], and Couto *et al*. [54] that demonstrated strong correlations between LE strip results and endometritis diagnosis using uterine lavage samples. Although slight variations in sensitivity and specificity have been reported across studies due to differences in sample types, cytological thresholds, and test brands, the cumulative evidence supports LE strip testing as a valuable, rapid, and non-invasive diagnostic method.

### Importance of combining diagnostic parameters

While single-parameter tests (pH or LE activity) exhibited substantial diagnostic value, their standalone performance was insufficient when CE and SCE were combined. Single markers may inadequately capture the nuanced inflammatory spectrum between subclinical and clinical disease, leading to under- or over-diagnosis.

By contrast, combining multiple parameters, particularly pH and LE strip results, enhanced diagnostic accuracy, sensitivity, and specificity. Moreover, combining these markers with traditional PVD assessment further improved diagnostic precision. The combined approach is not only diagnostically robust but also practical, as pH and LE strips are readily available, user-friendly, and easily integrated into routine cow-side assessments.

The simplicity of this method, coupled with its improved performance, renders it highly suitable for large-scale application in dairy herd management. Furthermore, the integration of artificial intelligence (AI) tools into the analysis of pH, LE activity, and additional parameters offers future avenues for automated and optimized diagnosis [55].

## CONCLUSION

This study demonstrated that the combined evaluation of vaginal discharge pH and LE activity offers a promising, practical approach for the cow-side diagnosis of endometritis in dairy cattle. Specifically, pH measurements obtained using a flat-surface electrode probe and pH and LE assessments through Combur10 Test® reagent strips showed significant diagnostic value, distinguishing cows with normal uterine health from those affected by CE and SCE. Optimal cut-off values of ≤8.35 for the pH meter, ≤8.0 for the pH strip, and ≥2 for the LE strip were established for diagnosing CE with good sensitivity and specificity. In contrast, TDS, salinity, and EC measurements lacked sufficient discriminative power for clinical application.

The practical implications of these findings are substantial: The use of pH and LE strips, either individually or in combination, provides a rapid, low-cost, and easily implementable tool for early detection of endometritis on farms. Adoption of such cow-side diagnostic methods may facilitate timely therapeutic interventions, thereby improving reproductive performance, reducing culling rates, and ultimately enhancing the economic sustainability of dairy operations.

A key strength of this study lies in the introduction and evaluation of a novel application of a flat-surface electrode probe for vaginal discharge analysis, as well as the systematic assessment of combined diagnostic parameters to enhance accuracy. The study design ensured consistency through the use of a single trained observer for clinical scoring and cytology evaluations, and the sample size provided adequate statistical power to detect significant diagnostic differences.

However, several limitations should be acknowledged. The relatively small and geographically limited study population may restrict generalizability to broader dairy systems. In addition, the requirement for sufficient discharge volume when using the pH meter may limit its practical use in some post-partum cows.

Future studies should aim to validate these findings across larger, more diverse cow populations and different management systems. Furthermore, direct assessment of endometrial pH and LE activity, integration with automated digital analysis, and exploration of AI-assisted interpretation could further refine and optimize cow-side diagnostic protocols. The development of portable, multiplex devices combining pH and LE detection may offer an even more streamlined solution for reproductive health monitoring in dairy herds.

In conclusion, pH and LE-based assessment of vaginal discharge presents a feasible, efficient, and scalable diagnostic approach for endometritis, with the potential to significantly improve reproductive health management in dairy cattle.

## AUTHOR’S CONTRIBUTIONS

NB: Conceptualized the study, methodology, data acquisition, data analysis, and drafted the manuscript. RJ: Data acquisition. SA: Data interpretation and critical review of the manuscript. KS and SS: Conceptualized the study and reviewed the manuscript. TS: Conceptualized the study, methodology, data acquisition and analysis, and drafted, reviewed, and edited the manuscript. All authors have read and approved the final version of the manuscript.
